# Dietary intervention in the treatment of patients with cough and symptoms suggestive of airways reflux as determined by Hull airways Reflux Questionnaire

**DOI:** 10.1186/1745-9974-9-27

**Published:** 2013-12-31

**Authors:** Joanna Elizabeth Smith, Jaymin Bhagwanji Morjaria, Alyn Hugh Morice

**Affiliations:** 1 Nutrition & Dietetics Department, Castle Hill Hospital, Hull & East Yorkshire NHS Trust, Castle Road, Cottingham, East Yorkshire HU16 5JQ, UK; 2 Respiratory Medicine, University of Hull & East Yorkshire Hospitals NHS Trust, Kingston upon Hull, UK

**Keywords:** Cough, Diet, Reflux, Quality of life, Weight

## Abstract

**Background:**

Chronic cough is a common and distressing symptom. Gastro-oesophageal reflux is a common cause of chronic cough however the symptom complex in cough is not confined to classic peptic symptoms. Dyspeptic symptoms have previously been shown to respond to dietary modifications and weight loss. We hypothesised that weight reduction maybe a useful non-pharmacological strategy in reducing reflux cough in the obese.

**Methods:**

Subjects with cough were recruited from Hull Cough Clinic. They were randomised to one of two open parallel groups; one receiving the traditional dietary modifications and the other weight reduction advice in the form of an Energy Prescription (EP). Cough symptoms, using the Leicester cough questionnaire (LCQ) and dietary intake were measured at the start and end of the study.

**Results:**

Thirty-three patients were recruited and 20 patients completed the study. Mean weight loss was 3.1 kg (p < 0.001) and reported an improvement in the LCQ (mean improvement 3.1); which is greater than the clinically significant score of 1.3. . Moreover, secondary outcomes showed a significant association between baseline high calorie (r = -0.24; p < 0.001) and fat intake (r = -0.36; p = 0.001), and LCQ scores.

**Conclusion:**

A high calorie and fat intake is strongly correlated with cough score. Irrespective of diet, weight loss is associated with a reduction in cough symptoms. Asking patients to lose weight by reducing fat and calorie intake may be a simple strategy to ameliorate this intractable condition.

**Trial Registration:**

The study was approved by the local research ethics committee (South Humber Local Research Ethics Committee; REC04/Q1105/62). The study was registered with the Research and Development Department, Clinical Governance Directorate, Hull and East Yorkshire Hospitals NHS Trust (reference number R0086).

## Background

Cough is the most common complaint leading patients to consult with primary care physicians [1,2]. Cough is a vital protective mechanism for clearing foreign material or excessive secretions from the airways. The common cold is the most frequent cause of cough in the acute (*i.e.*, <3 weeks in duration) setting and is usually self-limiting. However, a persistently troublesome chronic cough (*i.e.*, >8 weeks in duration) of obscure aetiology has been shown to be one of the most common reasons for new patient visits to respiratory physicians in secondary/tertiary care [3].

Gastro-oesophageal reflux is a common cause of chronic cough [4]. A number of studies have reported a higher incidence of classic acid related reflux symptoms (gastro-oesophageal reflux disease (GORD)) in overweight and obese people [5-8]. In a systematic review of 16 studies of GORD management, weight loss was associated with improved oesophageal pH profiles and GORD related symptoms [9]. We have recently demonstrated an association of a chronic cough with obesity [10] and have observed in clinical practice that weight gain can be associated with the onset of chronic cough. The effect of weight loss on cough has not been studied.

The traditional dietary advice given to GORD patients is not concerned with weight loss, but is centred around dietary modifications, for example avoidance of spicy foods, coffee, alcohol and high fat foods [11,12]. However recent studies have been unable to confirm the efficacy of such measures [9,13,14]. A systematic review concluded that there was insufficient data to support or refute common recommendations to change diet [15]. Thus the evidence base for such advice appears poor and the prevailing opinion is summarised by DeVault and Castell who suggest that traditional diet therapy ‘may benefit many patients with GORD, although these changes alone are unlikely to control symptoms in the majority of patients’ [16]. The current National Institute of Clinical Excellence (NICE) guidance is more dismissive “available trials of lifestyle advice to reduce symptoms of dyspepsia are small and inconclusive” and that there is “no clear association between dyspepsia and other lifestyle factors: smoking, alcohol, coffee, and diet” [17]. Thus there is a conflict between traditional dietary practice and the evidence for the role of conventional dietary intervention in GORD.

The form of reflux leading to cough is different in constitution, location and timing to that associated with classic GORD symptoms such as heartburn [18]. These reflux events may be extra-oesophageal, of a shorter duration and not necessarily acid in nature. The diagnosis of such airway reflux relies on the clinical history, but may be established by the use of validated, reproducible and responsive questionnaires such as the Hull Airways Reflux Questionnaire (HARQ) [19]. The differences between airway reflux and classic GORD symptoms has previously lead to diagnostic confusion with many patients being mislabelled as suffering from an asthmatic or idiopathic cough.

We hypothesised that weight reduction may be a useful non-pharmacological strategy for patients presenting with chronic cough. We therefore undertook a study comparing two dietetic strategies, weight loss and anti-reflux, in obese patients with a positive HARQ response indicating significant symptoms of airway reflux. As a weight loss strategy we used the energy prescription method (EP) as described by Frost et al. [20]. This was compared with the traditional GORD anti-reflux diet suggested within the Manual of Dietetic Practice [21] in an open parallel group study.

### Brief statement of findings

We assessed two different dietary regimens and whether they would help attenuate cough reflux. We found that weight reduction, irrespective of diet reduced cough reflux symptoms and this has not been found before.

## Methods

Patients were recruited from the Hull Cough Clinic at Castle Hill Hospital. The study was approved by the local research ethics committee (South Humber Local Research Ethics Committee; REC04/Q1105/62). The study was registered with the Research and Development Department, Clinical Governance Directorate, Hull and East Yorkshire Hospitals NHS Trust (reference number R0086). The study was completed between June 2005 and September 2009.

### Patients

Patients were diagnosed as have cough secondary to airway reflux based on the validated HARQ score of >13 [19]. This is represents the upper limit of normal on the self-completed, fourteen component instrument. Based on the WHO classification of obesity [22], patients with a body mass index (BMI) >25 kg/m^2^ were asked whether they wished to participate in a trial of dietary intervention. Following informed consent patients were randomised in a double-blinded manner using a Latin Square fashion to one of two open parallel groups.

### Intervention

One group received the traditional dietary modifications consisting of personalised advice concerning eating smaller meals at regular intervals; avoiding eating late at night; avoiding bending/lifting or lying down after meals, and general encouragement to lose weight by following a balanced diet plan [23]. Excessive consumption of tea/coffee/alcohol was to be avoided as was highly spiced or irritant foods or particular foods known to exacerbate symptoms. Sleeping in a semi-upright position or raising the head of the bed to help prevent nocturnal symptoms of reflux was encouraged [21].

The other group received dietary advice on weight reduction in the form of an EP diet [20]. The EP is calculated using the individual’s basal metabolic rate (BMR) based on age, gender and weight in kg, adding on a physical activity level (PAL) based on gender and activity level to then calculate estimated energy requirements (EER). The EER then has 600 kcal subtracted. An individualised balanced menu plan is then provided with guidance on portion sizes.

At baseline all patients had dietary and anthropometric assessments and were advised on the dietary treatment plan by a registered dietician (JS). Both groups were asked to complete the Leicester Cough Questionnaire [24] and 5 day food diaries at the beginning and end of the 6 month observation study period. All patients were reviewed at monthly intervals when compliance was assessed and dietary advice reinforced.

### Analyses

Baseline and demographic data were expressed as mean (± standard deviation (SD)) or median (interquartile range). Post-intervention data were also similarly expressed. Statistical comparisons of the effect of the interventions within and between groups were carried out using Wilcoxon-signed rank and Kruskal-Wallis test respectively. Due to the high drop out rate and inequality between the two groups modified intention to treat (ITT) analyses was performed. Missing observations were imputed using last outcome carried forward, though we note this approach is not without its limitations [25]. A two-tailed p value of less than 0.05 was considered to indicate statistical significance. All analyses were performed with the Statistical Package for Social Science (SPSS for windows version 18.0, Chicago, IL, USA). Correlation assessments were conducted and were expressed as R and p values. Food diary analysis was completed using the Microdiet© Plus for Windows (version 1.2, 2001)*.*

## Results

**Figure 1:**
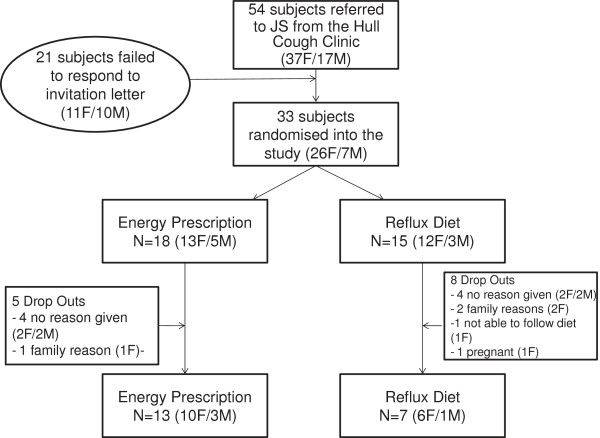
Flow diagram of the progress through the phases of the study.

The flow diagram in Figure 1 demonstrates the progress through the phases of the open parallel randomised trial for both groups. Thirty three patients (26 female) consented to take part in the study, mean age 56.7 years. Twenty subjects (16 female) with a mean age 58.6 years completed the study. At baseline they had a mean HARQ score of 30/70. Baseline parameters of the patients are depicted in Table 1 and the change in parameters from baseline are depicted in Table 2.

**Table 1:** Baseline parameters of all consented, energy prescription and reflux diet patients in the study

**Parameter**	**Consented patients (N = 33)**	**Energy prescription Gp patients (N = 18)**	**Reflux diet Gp patients (N = 15)**
Age*	58.6 (±13.4)	58.0 (±14)	59.0 (±13)
Sex	4 M/16 F	3 M/10 F	1 M/6 F
Baseline weight (Kg)*	89.0 (±17.4)	89.9 (±13.7)	87.5 (±24.0)
Baseline BMI (kg/m2)^	34.0 (29.8, 35.3)	33.6 (30.5, 34.9)	31.8 (28.5, 35.6)
Baseline LCQ score*	14.4 (±3.8)	6.8 (±2.6)	2.58 (±1.61)
Baseline Kcal^	1266.7 (1138.5, 1532.9)	1396.7 (1147.9, 1615.9)	1165.9 (1138.5, 1336.9)
Baseline fat^	41.8 (32.8, 57.1)	51.2 (31.6, 64.4)	41.4 (35.6, 65.6)

*Expressed as mean (± standard deviation).

^Expressed as median (interquartile range).

*Abbreviations:Gp* Group, *N* number, *M* male, *F* female, *Kg* kilograms, *BMI* body mass index, *kg/m2* kilogram per metres squared, *LCQ* Leicester cough questionnaire, *Kcal* Kilocalories.

**Table 2:** Change in parameters for the intention to treat cohorts for the energy prescription and reflux diets

**Parameter**	**Energy prescription diet**		**Reflux diet**	
	**Baseline**	**End of study**	**Baseline**	**End of study**
Baseline weight (Kg)*	89.9 (±13.7)	85.9 (±15.4)	87.5 (±24.0)	82.1 (±24.0)
Baseline BMI (kg/m2)^	33.6 (30.5, 34.9)	32.5 (28.3, 34.3)	31.8 (28.5, 35.6)	29.7 (26.1, 34.4)
Baseline LCQ score*	6.8 (±2.6)	6.2 (±2.5)	2.58 (±1.61)	13.5 (±3.7)
Baseline Kcal^	1396.7 (1147.9, 1615.9)	1493.4 (1147.9, 1615.9)	1165.9 (1138.5, 1336.9)	1400.4 (1276.9, 1552.0)
Baseline fat^	51.2 (31.6, 64.4)	53.7 (48.6, 64.4)	41.4 (35.6, 65.6)	48.7 (47, 71.2)

*Expressed as mean (± standard deviation).

^Expressed as median (interquartile range).

*Abbreviations:Gp* Group, *N* number, *M* male, F female, *Kg* kilograms, *BMI* body mass index, *kg/m2* kilogram per metres squared, *LCQ* Leicester cough questionnaire, *Kcal* Kilocalories.

Mean weight loss was 3.1 kg representing a 0.4% (range -3 – 11%) weight loss of all patients (p < 0.001). All but two completing patients lost weight. Mean weight loss in patients completing the programme was 3.9%. In both intervention arms there was a similar and significant weight loss. For the EP group mean reduction was 3.2 kg (p = 0.001), and for the anti-reflux diet group a mean reduction of 2.63 kg (p = 0.012).

**Figure 2:**
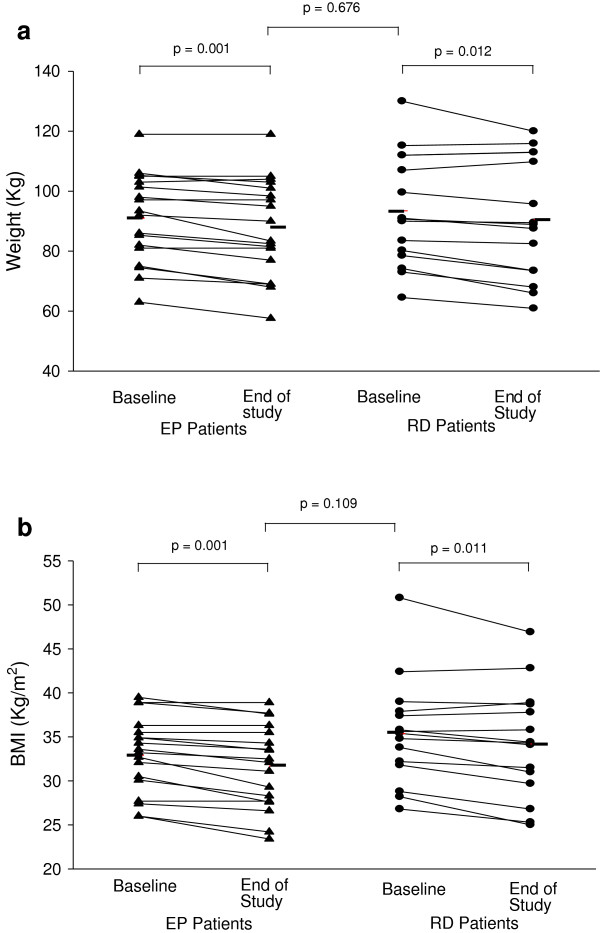
**Change in weight and BMI from baseline to end of study. (a)** weight (kg). **(b)** BMI (kg/m2).

At baseline, mean BMI 34 kg/m^2^ (range 26-50.8 kg/m^2^). There were similar and significant changes from baseline to end of study for the EP group (mean reduction 1.3 kg/m^2^ (p = 0.001)), and for the anti-reflux diet group (mean reduction 1.2 kg/m^2^ (p = 0.011)) (Table 1; Figures 2a, 2b).

Sixteen patients submitted food diaries at both the start and end of the trial. 10 of these subjects were in the EP group. Surprisingly for both groups the food diary analysis from baseline to end of study, showed an increase in both calorie and fat intakes, though these increases were not significant. For the EP group the mean increase in calories consumed was 96.7 kcal (p = 0.4) and for the anti-reflux diet group was 234.5 kcal (p = 1.0). The mean increase in fat intake for the EP group was 2.5 g (p = 0.8) and for the anti-reflux diet group was 7.3 g (p = 0.8) (Table 2).

**Figure 3:**
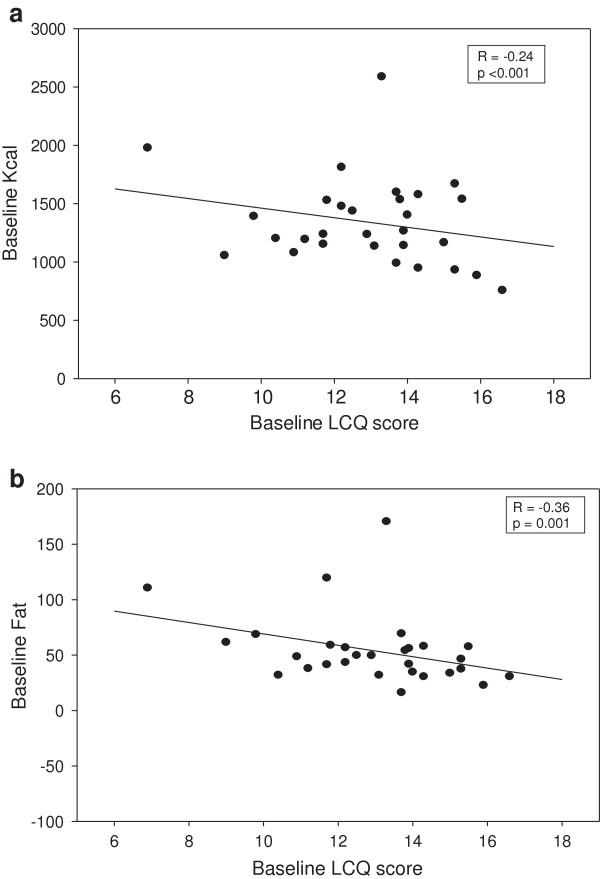
**Correlations of calorie and fat intakes with LCQ score at baseline, (a)** calorie (kcal). **(b)** fat (g).

The mean change in LCQ score was 3.1 units in all the consented patients. There was an increase (improvement in LCQ) was similar (3.6 units for the EP group and 2.5 units for the anti-reflux diet group) (Table 2). All of the patients who completed the study achieved an improvement in their cough score (range 2.5 to 7.7) above the minimal clinically significant (MCS) score of 1.3 [26]. Secondary correlation analysis of the baseline data from the food diary showed that high calorie and fat intakes were significantly associated with a worse quality of life score (calories (r = -0.24; P < 0.001); fat (r = -0.36; P = 0.001)) (Figure 3a, 3b).

## Discussion

Chronic cough associated with gastro oesophageal reflux is a common but frequently under diagnosed condition. This is due to the ‘atypical’ presentation with symptoms of non-peptic, extra-oesophageal origin. To define this syndrome a variety of questionnaires such as the Reflux Symptom Index have been developed. In this study we have used the HARQ, a questionnaire developed specifically to quantify the symptoms of airway reflux underlying cough hypersensitivity. Our patients all exhibited HARQ scores above the cut off of 13/70 required to diagnose airway reflux and represent a distinctive population of patients with reflux induced cough hypersensitivity syndrome [19].

There is a dearth of treatment options available to patients with airway reflux. Pharmacotherapy with conventional acid suppression has recently been shown to be no more effective than placebo [27] presumably because of the non-peptic nature of the tussive stimulus. Pro-motility agents such as metoclopramide, domperidone and macrolides have been advocated, but there is a lack of randomised control trials supporting their use. The only evidence based therapy is oral opiates [28], which do not treat reflux but suppresses cough reflex hypersensitivity centrally.

Non-pharmacological treatment has been shown to be effective. Specific cough related speech therapy has been demonstrated to diminish cough reflex sensitivity and improve quality of life [29]. We had previously observed a significant relationship between obesity and chronic cough in a survey of the general population [10]. This observation is mirrored in studies showing an increase of obesity related GORD [30]. This led us to hypothesize that weight reduction may offer a further non-pharmacological treatment option in obese chronic cough patients.

We have tested this hypothesis by comparing two dietary measures that may aid resolution of airway reflux. The traditional ‘textbook’ GORD anti-reflux diet was compared to an EP diet, targeting weight control. We showed no difference between these two strategies, both of which produced weight loss and a reduction in cough score. Surprisingly, percentage weight loss was slightly greater in the traditional ‘textbook’ GORD anti-reflux diet group, and there appears to be little evidence that dietary intervention needs to follow a restriction of foods of any particular ‘refluxogenic’ type for improvement to be manifest.

Analyses of the food diaries at baseline showed current high intakes of calories and fat had an adverse effect on cough quality of life suggesting that this may contribute to chronic cough. Other studies in GORD have demonstrated an association of a high fat intake and the occurrence of reflux [11,12]. Moreover, weight loss has been shown to improve pH profiles and symptoms in GORD patients [9]. An alternative mechanism to explain our observation may be that the high fat and calorie intake leads to weight gain which in turn causes reflux. This is supported by research suggesting that reflux is more prevalent in the overweight and obese population rather than the lean [5-8].

Since there was no non-intervention group in our study it is impossible to say whether the dietary measures directly contributed to the improvement in cough quality of life. Chronic cough may follow a waxing and waning course [31] and it is possible that the improvement in cough score may be explicable by regression to the mean from the pre study baseline. The effectiveness of a dietary intervention on cough however, with all of the patients who completed the study showing a greater than MCS improvement in cough quality of life score, would tend to suggest a clinically important effect of this intervention.

In interpreting our observations one needs to take into account some limitations in our study. Firstly, recruitment to the study was slower than expected due to 21 patients who declined to take part from the outset despite encouragement from the medical team. Patients in the Hull Cough Clinic often request advice on lifestyle modification and it was perhaps surprising that these proposals were rejected when offered. There are many factors that contribute to a person’s motivation to lose weight which include their readiness for change and barriers to change such as how diet and exercise affects health. As our study required that the patient to be reviewed monthly over a seven month period, study duration may have been a contributing factor. An individual’s perceived lack of time is one of the barriers to change [32]. NICE Guidelines on Obesity however, recommends that ‘regular, non-discriminatory long term follow up by a trained professional should be offered’ [32]. It is possible that these recommendations may hamper rather than aid compliance. Secondly, as with many studies into the effect of lifestyle change, this study could not use a blinded methodology. As a consequence all patients recruited were aware of our hypothesis that weight loss would contribute to a reduction in reflux cough. Thirteen of the recruited subjects dropped out of the study, with 8 subjects providing no reason. A number who completed the study stated that they were disappointed to be allocated into what was perceived as the control group, i.e. the traditional anti-reflux diet. Moreover, the overall number of patients who completed the study was small. Thirdly, we did not perform objective assessments of GORD since existing techniques are poorly correlated with non acid reflux as determined by symptom assessment. There is an urgent need for such measurements to be developed. Additionally we did not study objective parameters of cough and we acknowledge this as a weakness of the study. The above may have diminished our understanding of the pathobiological basis of the cough reduction seen. In particular, Obstructive Sleep Apnoea (OSA) in obese subjects can rarely precipitate cough [33], and maybe improved by weight reduction. While we have shown statistical significance effects however, studies on larger numbers of patients will be required to draw definitive conclusions.

## Conclusion

In this study we report for the first time if weight loss is achieved irrespective of diet there is a reduction on cough quality of life symptoms. Furthermore, we have shown a strong correlation between a high calorie and fat intake with LCQ score. Simply asking subjects to lose weight by reducing fat and calorie intake may be a useful strategy for the management of intractable cough in primary care.

## Abbreviations

BMR: Basal metabolic rate; EER: Estimated energy requirements; EP: Energy prescription; GORD: Gastro-oesophageal reflux disease; HARQ: Hull airways reflux Questionnaire; ITT: Intention to treat; LCQ: Leicester cough questionnaire; MCS: Minimally clinically significant; NICE: National institute of clinical excellance; NHS: National health service; OSA: Obstructive sleep apnoea; PAL: Physical activity level; SPSS: Statistical package for social science; WHO: World Health Organisation.

## Competing interests

The authors declare that they have no competing interests in relation to this article. No funding or sponsorship was received for this study.

## Authors’ contributions

Patients for the study were selected by AHM; dietary advice, running of the study and food diary analyses were conducted by JES; and the writing of the manuscript was equally contribute by JES, JBM and AHM. All authors read and approved the final manuscript.

## References

[B1] Morrell DC Symptom interpretation in general practice J R Coll Gen Pract 1972 9 297 309 5073371 PMC2156732

[B2] Britt H Miller GC Knox S Charles J Valenti L Henderson J Pan Y Sutton C Harrison C General practice activity in Australia 2001-2 Book General practice activity in Australia 2001-2.(Editor ed.^eds.), vol. General Practice seris No.10, AIHW Cat. GEP10 edition 2002 Canberra: Australian Instiute of Health and Welfare

[B3] Irwin RS Introduction to the diagnosis and management of cough: ACCP evidence-based clinical practice guidelines Chest 2006 9 25S 27S 10.1378/chest.129.1_suppl.25S 16428688

[B4] Morice AH Chronic cough: epidemiology Chron Respir Dis 2008 9 43 47 10.1177/1479972307084252 18303101

[B5] Mathus-Vliegen EM Tygat GN Gastro-oesophageal reflux in obese subjects: influence of overweight, weight loss and chronic gastric balloon distension Scand J Gastroenterol 2002 9 1246 1252 10.1080/003655202761020498 12465720

[B6] Murray L Johnston B Lane A Harvey I Donovan J Nair P Relationship between body mass and gastro-oesophageal reflux symptoms: the Bristol Helicobacter Project Int J Epidemiol 2003 9 645 650 10.1093/ije/dyg108 12913045

[B7] Locke GR 3rd Talley NJ Fett SL Zinsmeister AR Melton LJ 3rd Risk factors associated with symptoms of gastroesophageal reflux Am J Med 1999 9 642 649 10.1016/S0002-9343(99)00121-7 10378622

[B8] Mercer CD Wren SF DaCosta LR Beck IT Lower esophageal sphincter pressure and gastroesophageal pressure gradients in excessively obese patients J Med 1987 9 135 146 3480930

[B9] Kaltenbach T Crockett S Gerson LB Are lifestyle measures effective in patients with gastroesophageal reflux disease? An evidence-based approach Arch Intern Med 2006 9 965 971 10.1001/archinte.166.9.965 16682569

[B10] Ford AC Forman D Moayyedi P Morice AH Cough in the community: a cross sectional survey and the relationship to gastrointestinal symptoms Thorax 2006 9 975 979 10.1136/thx.2006.060087 16809412 PMC2121176

[B11] El-Serag HB Satia JA Rabeneck L Dietary intake and the risk of gastro-oesophageal reflux disease: a cross sectional study in volunteers Gut 2005 9 11 17 10.1136/gut.2004.040337 15591498 PMC1774352

[B12] Kitchin LI Castell DO Rationale and efficacy of conservative therapy for gastroesophageal reflux disease Arch Intern Med 1991 9 448 454 10.1001/archinte.1991.00400030018004 1672062

[B13] Boekema PJ Samsom M Smout AJ Effect of coffee on gastro-oesophageal reflux in patients with reflux disease and healthy controls Eur J Gastroenterol Hepatol 1999 9 1271 1276 10.1097/00042737-199911000-00015 10563539

[B14] Penagini R Mangano M Bianchi PA Effect of increasing the fat content but not the energy load of a meal on gastro-oesophageal reflux and lower oesophageal sphincter motor function Gut 1998 9 330 333 10.1136/gut.42.3.330 9577336 PMC1727046

[B15] Chang AB Lasserson TJ Kiljander TO Connor FL Gaffney JT Garske LA Systematic review and meta-analysis of randomised controlled trials of gastro-oesophageal reflux interventions for chronic cough associated with gastro-oesophageal reflux BMJ 2006 9 11 17 10.1136/bmj.38677.559005.55 16330475 PMC1325125

[B16] DeVault KR Castell DO Updated guidelines for the diagnosis and treatment of gastroesophageal reflux disease Am J Gastroenterol 2005 9 190 200 10.1111/j.1572-0241.2005.41217.x 15654800

[B17] National Institute of Clinical Excellence Dyspepsia – Management of dyspepsia in adults in primary care Natl Inst Clin Excell 2004 9 1 47

[B18] Everett CF Morice AH Clinical history in gastroesophageal cough Respir Med 2007 9 345 348 10.1016/j.rmed.2006.05.006 16787744

[B19] Morice AH Faruqi S Wright CE Thompson R Bland JM Cough hypersensitivity syndrome: a distinct clinical entity Lung 2011 9 73 79 10.1007/s00408-010-9272-1 21240613

[B20] Frost G Masters K King C Kelly M Hasan U Heavens P White R Stanford J A new method of energy prescription to improve weight loss J Hum Nutr Diet 1991 9 369 373 10.1111/j.1365-277X.1991.tb00120.x 17539863

[B21] Thomas B, Bishop J Manual of Dietetic Practice 4th Edition – Section 1.5 (Dietetic Assessment); Section 4.4 (Disorders of the stomach and duodenum); Section 4.16 (Obesity – General Aspects); Section 4.17 (Management of obesity and overweight) 2007 4 Oxford: Blackwell Publishing

[B22] Obesity Preventing and managing the global epidemic Book Obesity. Preventing and Managing the Global Epidemic 1998 11234459

[B23] The Balance of Good Health: Information for educators and communicators 2001 Food Standards Agency http://www.foodstandards.gov.uk

[B24] Birring SS Prudon B Carr AJ Singh SJ Morgan MD Pavord ID Development of a symptom specific health status measure for patients with chronic cough: Leicester Cough Questionnaire (LCQ) Thorax 2003 9 339 343 10.1136/thorax.58.4.339 12668799 PMC1746649

[B25] Molenberghs G What to do with missing data? J R Stat Soc Ser A 2007 9 861 863 [Editorial] 10.1111/j.1467-985X.2007.00504.x

[B26] Raj AA Pavord DI Birring SS Clinical cough IV:what is the minimal important difference for the Leicester Cough Questionnaire? Handb Exp Pharmacol 2009 9 311 320 10.1007/978-3-540-79842-2_16 18825348

[B27] Faruqi S Molyneux ID Fathi H Wright C Thompson R Morice AH Chronic cough and esomeprazole: a double-blind placebo-controlled parallel study Respirology 2011 9 1150 1156 10.1111/j.1440-1843.2011.02014.x 21707852

[B28] Morice AH Menon MS Mulrennan SA Everett CF Wright C Jackson J Opiate therapy in chronic cough Am J Respir Crit Care Med 2007 9 312 315 10.1164/rccm.200607-892OC 17122382

[B29] Vertigan AE Theodoros DG Gibson PG Winkworth AL Efficacy of speech pathology management for chronic cough: a randomised placebo controlled trial of treatment efficacy Thorax 2006 9 1065 1069 10.1136/thx.2006.064337 16844725 PMC2117063

[B30] El-Serag H The association between obesity and GERD: a review of the epidemiological evidence Dig Dis Sci 2008 9 2307 2312 10.1007/s10620-008-0413-9 18651221 PMC2827866

[B31] Chung KF Pavord ID Prevalence, pathogenesis, and causes of chronic cough Lancet 2008 9 1364 1374 10.1016/S0140-6736(08)60595-4 18424325

[B32] Obesity Clinical Guideline 43 guidance on the prevention, identification, assessment and management of overweight and obesity in adults and children: clinical guideline 43 Book Obesity: guidance on the prevention, identification, assessment and management of overweight and obesity in adults and children 2006 22497033

[B33] Faruqi S Fahim A Morice AH Chronic cough and obstructive sleep apnoea: reflux-associated cough hypersensitivity? Eur Respir J 2012 9 4 1049 1050 10.1183/09031936.00025012 23024327

